# Vascular endothelial growth factor regulates myeloid cell leukemia-1 expression through neuropilin-1-dependent activation of c-MET signaling in human prostate cancer cells

**DOI:** 10.1186/1476-4598-9-9

**Published:** 2010-01-19

**Authors:** Shumin Zhang, Haiyen E Zhau, Adeboye O Osunkoya, Shareen Iqbal, Xiaojian Yang, Songqing Fan, Zhengjia Chen, Ruoxiang Wang, Fray F Marshall, Leland WK Chung, Daqing Wu

**Affiliations:** 1 Department of Urology and Winship Cancer Institute, Emory University School of Medicine, Atlanta, GA, USA; 2 Uro-Oncology Research Program, Cedars-Sinai Medical Center, Los Angeles, CA, USA; 3 Department of Pathology & Laboratory Medicine, Emory University School of Medicine, Atlanta, GA, USA; 4 Department of Urology, Xijing Hospital, Fourth Military Medical University, Xi'an, China; 5 Department of Pathology, the Second Xiangya Hospital, Central South University, Changsha, China; 6 Department of Biostatistics and Bioinformatics, Rollins School of Public Health, Emory University, Atlanta, GA, USA

## Abstract

**Background:**

Myeloid cell leukemia-1 (Mcl-1) is a member of the Bcl-2 family, which inhibits cell apoptosis by sequestering pro-apoptotic proteins Bim and Bid. Mcl-1 overexpression has been associated with progression in leukemia and some solid tumors including prostate cancer (PCa). However, the regulatory mechanism for Mcl-1 expression in PCa cells remains elusive.

**Results:**

Immunohistochemical analyses revealed that Mcl-1 expression was elevated in PCa specimens with high Gleason grades and further significantly increased in bone metastasis, suggesting a pivotal role of Mcl-1 in PCa metastasis. We further found that vascular endothelial growth factor (VEGF) is a novel regulator of Mcl-1 expression in PCa cells. Inhibition of endogenous Mcl-1 induced apoptosis, indicating that Mcl-1 is an important survival factor in PCa cells. Neuropilin-1 (NRP1), the "co-receptor" for VEGF_165 _isoform, was found to be highly expressed in PCa cells, and indispensible in the regulation of Mcl-1. Intriguingly, VEGF_165 _promoted physical interaction between NRP1 and hepatocyte growth factor (HGF) receptor c-MET, and facilitated c-MET phosphorylation *via *a NRP1-dependent mechanism. VEGF_165 _induction of Mcl-1 may involve rapid activation of Src kinases and signal transducers and activators of transcription 3 (Stat3). Importantly, NRP1 overexpression and c-MET activation were positively associated with progression and bone metastasis in human PCa specimens and xenograft tissues.

**Conclusions:**

This study demonstrated that Mcl-1 overexpression is associated with PCa bone metastasis. Activation of VEGF_165_-NRP1-c-MET signaling could confer PCa cells survival advantages by up-regulating Mcl-1, contributing to PCa progression.

## Background

Acquisition of apoptosis resistance is characteristic of invasive tumor cells. Elevated expression of anti-apoptotic proteins is associated with tumor progression clinically and experimentally [1]. Myeloid cell leukemia-1 (Mcl-1), a member of the Bcl-2 family, sequesters pro-apoptotic proteins Bim and Bid, thereby inhibiting mitochondrial outer membrane permeabilization, a central control point of apoptosis [2,3]. Mcl-1 overexpression is associated with progression in leukemia [4] and some solid tumors including prostate cancer (PCa) [5-7]. Mcl-1 was elevated in primary PCa with high Gleason grades and metastatic tumors compared to that in prostatic intraepithelial neoplasia (PIN) or lower grade tumors, suggesting a pivotal role of Mcl-1 in PCa progression [5].

Angiogenesis favors tumor cell survival, thereby contributing to progression [1]. Vascular endothelial growth factor (VEGF) is a critical pro-angiogenic factor that induces proliferation and migration of endothelial cells within tumor vasculature [8]. VEGF is expressed as several alternately spliced isoforms. VEGF_165 _is pre dominant, with optimal bioavailability, and responsible for VEGF biological potency, whereas VEGF_121 _is less potent but freely diffusible. VEGF binds two highly-related receptor tyrosine kinases, VEGF-R1 and VEGF-R2 [8]. Neuropilin-1 (NRP1) was originally identified as a receptor for the semaphorin 3 subfamily mediating neuronal guidance and axonal growth [9]. It was subsequently found to specifically bind VEGF_165 _but not VEGF_121 _on endothelial cells and tumor cells [9,10]. NRP1 lacks a typical kinase domain, primarily functioning as a "co-receptor" to form ligand-specific receptor complexes. In response to VEGF_165_, NRP1 couples with VEGF-Rs to signal in endothelial cells. Though VEGF-R1 and VEGF-R2 are usually absent or expressed at very low levels in PCa cells [11], aberrant upregulation of NRP1 has been frequently observed in high grade and metastatic PCa and other solid tumors [9,12-16]. Ectopic expression of NRP1 in PCa cells induced cell migration, increased tumor size and microvessel density, and inhibited apoptosis [17]. These observations suggested that NRP1 may be critical for PCa progression. Nonetheless, the mechanism by which NRP1 transmits VEGF signaling in PCa cells lacking VEGF-Rs remains unclear.

Previously we reported that serum VEGF levels correlate to bone metastatic status in PCa patients, and activation of VEGF signaling in PCa cells is associated with invasive phenotypes in experimental models [18]. In this study, we correlate Mcl-1 overexpression to PCa progression towards bone metastasis, and provide evidence that VEGF regulates Mcl-1 expression through NRP1-dependent activation of c-MET in PCa cells.

## Results

### Elevated Mcl-1 expression is associated with PCa progress and bone metastasis

**Figure 1:**
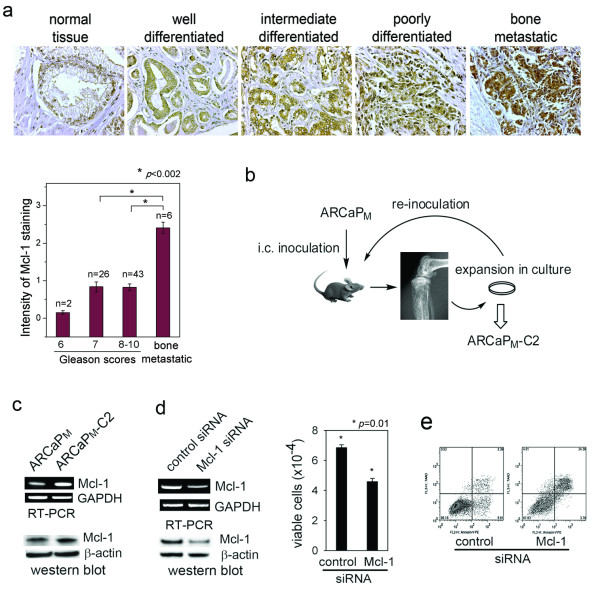
**Mcl-1 is a survival factor in human PCa cells**. (a) IHC staining of Mcl-1 in human PCa tissue microarray consisting of normal adjacent tissues, primary PCa and bone metastases. (b) Intracardiac (i.c) injection of ARCaP_M _cells in athymic mice resulted in metastases to bone and soft tissues. The ARCaP_M_-C2 subclone was derived from metastatic bone tissues after two rounds of intracardiac inoculation of ARCaP_M _cells. (c) Endogenous Mcl-1 expression in the ARCaP_M _model was examined by RT-PCR and western blotting analyses. (d) Effect of Mcl-1 siRNA on ARCaP_M _cell viability. Subconfluent ARCaP_M _cells on a 6-well plate were transiently transfected with Mcl-1 siRNA (30 nM) for 72 h. Endogenous expression of Mcl-1 at the mRNA and protein levels, and cell viability of ARCaP_M _cells as counted with trypan blue staining, were significantly inhibited by Mcl-1 siRNA treatment compared to the control. (e) Effects of Mcl-1 siRNA on ARCaP_M _cell apoptosis. Subconfluent ARCaP_M _cells were transfected with Mcl-1 siRNA or control siRNA for 72 h, expression of annexin V was measured by FACS.

To investigate the clinical significance of Mcl-1 in PCa progression, immunohistochemical (IHC) analyses were performed to determine the expression of Mcl-1 in a human PCa tissue microarray with matched normal adjacent tissue and bone metastatic bone specimens (Figure 1a). We defined tumors with Gleason score 2-6 as well-differentiated (n = 2), Gleason score 7 as intermediately-differentiated (n = 26) and Gleason score 8-10 as poorly-differentiated (n = 43). Mcl-1 immunointensity was increased from normal tissues to well-differentiated cancer and further elevated in high grade PCa, although the difference between Mcl-1 intensity in intermediate- and poorly-differentiated cancers was not statistically significant (*p *= 0.93). Intriguingly, Mcl-1 staining in bone metastatic tumors (n = 6) was remarkably increased compared to that in either intermediate- or poorly-differentiated PCa (*p *< 0.002). These data correlated elevated Mcl-1 expression to clinical PCa progression, particularly bone metastasis.

### Mcl-1 is a survival factor in human PCa cells

We have established several lines of human PCa models that closely mimic the clinical progression of PCa bone metastasis, including the ARCaP model [19,20]. When inoculated either orthotopically or intracardially, ARCaP_M _cells are capable of metastasizing spontaneously to bone and soft tissues, forming mixed osteoblastic and osteolytic lesions in mouse skeleton [19,21,22]. The ARCaP_M_-C2 subclone, derived from metastatic bone tissues after two rounds of intracardiac injection of ARCaP_M _cells in athymic mice, forms predictable metastases to bone, adrenal gland and other soft tissue with higher propensity and shorter latency [21,22] (Figure 1b). Reverse transcription-PCR (RT-PCR) and immunoblotting analyses found that Mcl-1 was substantially expressed by ARCaP_M _cells, and further increased in ARCaP_M_-C2 cells (Figure 1c). Similarly, increased Mcl-1 expression was observed in metastatic C4-2 and C4-2B cells [23] when compared to their parental, androgen-dependent human PCa cell line LNCaP (Additional file 1, Figure S1a). These results suggested a possible association between Mcl-1 expression and invasive phenotypes of PCa cells.

To examine the function of Mcl-1 in PCa cell survival, a Mcl-1 small-interfering RNA (siRNA) was transfected into ARCaP_M _cells. siRNA treatme nt effectively inhibited Mcl-1 expression and significantly reduced ARCaP_M _cell viability by ~36% after 72 h (Figure 1d). Annexin V staining by fluorescence-activated cell sorting (FACS) analysis showed that the Mcl-1 siRNA induced apoptosis in 24.4-2.3% of ARCaP_M _cells (Figure 1e). These results indicated that Mcl-1 may be an important survival factor in PCa cells.

### VEGF regulates Mcl-1 expression in human PCa cells

**Figure 2:**
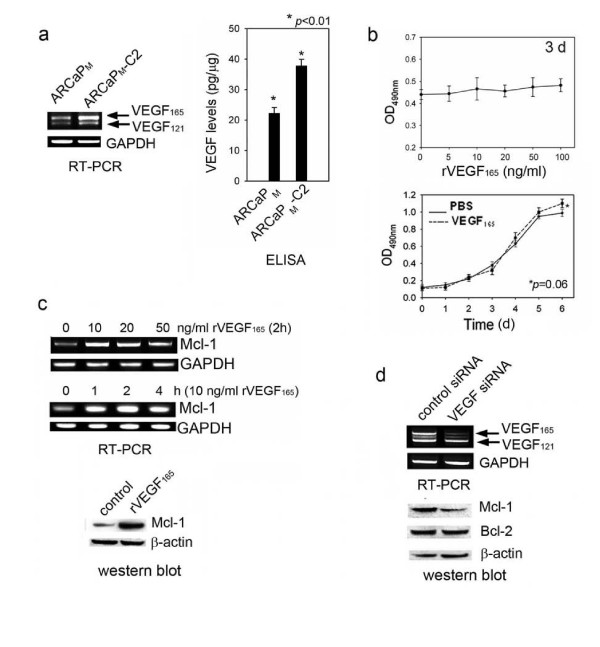
**VEGF_165 _regulates Mcl-1 expression in PCa cells**. (a) VEGF expression in ARCaP_M _and ARCaP_M_-C2 cells, as determined by RT-PCR and ELISA (conditioned medium). (b) Effects of recombinant VEGF_165 _on cell proliferation as determined by MTS assay. Top, ARCaP_M _cells were seeded in 96-well plates (1 × 10^3^cells/well) for 24 h, and serum-starved overnight. The cells were further incubated with varying concentrations of VEGF_165 _in serum-free T-medium for 72 h. Bottom, ARCaP_M _cells were incubated with PBS or VEGF_165 _(50 ng/ml) in serum-free T-medium for varying times. (c) VEGF_165 _effects on Mcl-1 expression. ARCaP_M _cells were treated with VEGF_165 _at indicated concentrations and times, and Mcl-1 mRNA expression was measured. For immunoblotting, ARCaP_M _cells were incubated with VEGF_165 _(10 ng/ml) for 72 h. (d) Effects of VEGF siRNA on Mcl-1 expression. ARCaP_M _cells were transfected with VEGF siRNA or control siRNA (80 nM) for 72 h.

Previously we reported that ARCaP_M _cells express high levels of endogenous VEGF, regulated by a cyclic AMP-response element binding protein (CREB)-hypoxia-inducible factor (HIF)-dependent mechanism in normoxic conditions [18]. In the present study, RT-PCR assay that could differentiate mRNA expression of VEGF_165 _and VEGF_121 _isoforms [18] was performed in ARCaP_M _and ARCaP_M_-C2 cells, showing a significant increase in the expression of both VEGF isoforms in ARCaP_M_-C2 cells, as confirmed at protein level by enzyme-linked immunosorbent assay (ELISA) (Figure 2a). Similarly, C4-2 cells express higher levels of VEGF when compared to LNCaP cells (Additional file 1, Figure S1b). These data suggested that VEGF expression is elevated in metastatic PCa cells.

VEGF potently stimulates endothelial cell proliferation and migration, an underlying mechanism for angiogenesis during tumor progression. However, neither exogenous human VEGF_165 _(Figure 2b) nor VEGF_121 _(Additional file 1, Figure S1c) significantly affected the proliferation of ARCaP_M _cells at a non-saturating range (5-100 ng/ml), suggesting that VEGF is not a potent mitogen in PCa cells. Intriguingly, VEGF_165 _was found to rapidly induce Mcl-1 mRNA expression in a dose- and time-dependent manner, with the maximum accumulation of Mcl-1 mRNA after 2 h-incubation at the concentration of 10 ng/ml. Western blot analysis confirmed a remarkable increase of Mcl-1 protein in ARCaP_M _cells (Figure 2c) and LNCaP cells (Additional file 1, Figure S1d) treated with VEGF_165_. Conversely, when ARCaP_M _cells were transfected with a VEGF siRNA nucleotide that selectively inhibited expression of VEGF_165 _but not VEGF_121_, expression of Mcl-1, but not Bcl-2, was reduced after 72 h (Figure 2d). Notably, Mcl-1 expression was not affected by VEGF_121 _treatment (Additional file 1, Figure S1e). These results indicated that VEGF_165 _may specifically regulate Mcl-1 expression in PCa cells.

### Differential expression of VEGF-Rs in PCa cells

**Figure 3:**
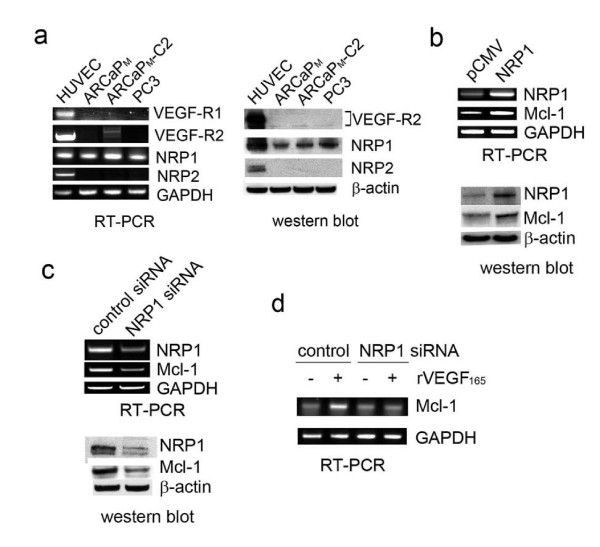
**NRP1 is required for basal expression and VEGF_165 _induction of Mcl-1 in ARCaP_M _cells**. (a) Expression of VEGF-Rs in PCa cells and HUVEC. (b) Effect of ectopic expression of NRP1 on Mcl-1 basal level. Subconfluent ARCaP_M _cells on 6-well plates were transfected with pCMV-NRP1 or pCMV-XL4 (16 μg) for 48 h (for RT-PCR) or 72 h (for immunoblotting). (c) Effects of NRP1 siRNA on Mcl-1 basal expression. ARCaP_M _cells were transfected with NRP1 siRNA or control siRNA (60 nM) for 48 h (for RT-PCR) or 72 h (for immunoblotting). (d) Effects of NRP1 siRNA on VEGF_165 _induction of Mcl-1. ARCaP_M _cells were transfected with NRP1 siRNA or control siRNA for 48 h, respectively. Cells were then serum-starved overnight, and treated with VEGF_165 _(10 ng/ml) or PBS for 2 h.

Expression of VEGF-Rs was examined in several PCa cell lines with human umbilical vein endothelial cells (HUVEC) as a positive control (Figure 3a, and Additional file 1, Figure S1f). VEGF-R1 was undetectable in PCa cells. Only very low expression of VEGF-R2 could be observed in ARCaP_M_-C2 cells. However, NRP1 was ubiquitously expressed in PCa cells at a level comparable to that in HUVEC, and higher in metastatic ARCaP_M_-C2, PC3, C4-2 and C4-2B cells. NRP2, the "co-receptor" for VEGF-C [8], was not detected in PCa cells. These data implied that NRP1 may be the major receptor mediating VEGF effects in PCa cells.

### NRP1 regulates both basal expression and VEGF induction of Mcl-1 in PCa cells

The role of NRP1 in the regulation of Mcl-1 expression was investigated. ARCaP_M _cells cultured in serum-containing T-medium were transiently transfected with a NRP1 expression vector. Compared to the control, ectopic expression of NRP1 resulted in increased Mcl-1 at both the mRNA and protein levels (Figure 3b). Conversely, transfection with a NRP1 siRNA specifically inhibited NRP1 and reduced endogenous Mcl-1 expression in ARCaP_M _cells (Figure 3c). These data indicated that NRP1 may be required and sufficient for basal expression of Mcl-1 in PCa cells. Further, ARCaP_M _cells were transfected with NRP1 siRNA or control siRNA, and incubated with VEGF_165 _in serum-free T-medium for indicated time. Figure 3d showed that expression of NRP1 siRNA, but not control siRNA, abrogated VEGF_165 _induction of Mcl-1 in ARCaP_M _cells. These data indicated an indispensible role of NRP1 in mediating VEGF_165 _induction of Mcl-1 in PCa cells.

### c-MET signaling is required for VEGF regulation of Mcl-1 in PCa cells

Since NRP1 does not contain typical kinase receptor sequences [24], we hypothesized that NRP1 may interact with certain tyrosine kinase receptor(s) to transmit VEGF autocrine signal in PCa cells lacking VEGF-Rs. Two recent studies independently demonstrated that NRP1 physically binds c-MET, and potentiates c-MET activation in response to HGF stimulation in human glioma and pancreatic cancer cells [25,26]. It is therefore plausible that c-MET may be involved in VEGF regulation of Mcl-1 in PCa cells. Indeed, HGF activation of c-MET signaling has been shown to transcriptionally increase Mcl-1 expression in primary human hepatocytes [27].

**Figure 4:**
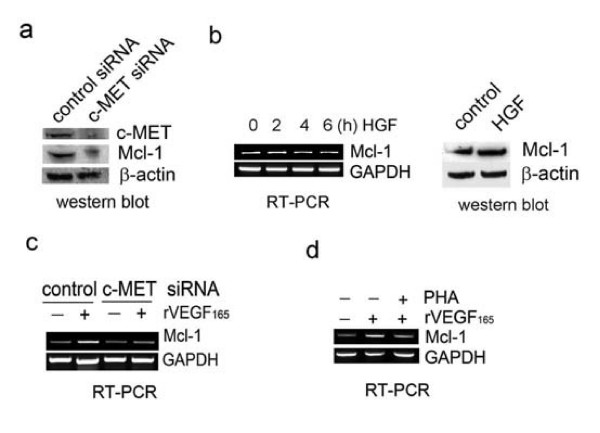
**c-MET signaling is required for VEGF_165 _induction of Mcl-1 in ARCaP_M _cells**. (a) Effects of c-MET inhibition on Mcl-1 expression. ARCaP_M _cells were transfected with c-MET siRNA or control siRNA (30 nM) for 72 h. (b) Effects of recombinant HGF treatment (10 ng/ml) on Mcl-1 expression at RNA (0, 2, 4 and 6 h) and protein levels (72 h). (c) ARCaP_M _cells were transfected with c-MET siRNA or control siRNA for 48 h, serum-starved overnight, and treated with VEGF_165 _(10 ng/ml) for 2 h. (d) ARCaP_M _cells were treated with PHA-665752 (0.5 μM) or dimethyl sulfoxide (DMSO) for 2 h, before treatment with VEGF_165 _(10 ng/ml) or PBS for 2 h.

A c-MET siRNA construct was transfected into ARCaP_M _cells, which effectively inhibited endogenous c-MET (Figure 4a). c-MET siRNA treatment reduced Mcl-1 protein expression, suggesting that c-MET is involved in maintaining basal expression of Mcl-1 in PCa cells. Interestingly, however, recombinant HGF treatment did not significantly affect Mcl-1 expression at either RNA or protein levels (Figure 4b), indicating that HGF-dependent activation of c-MET signaling is not sufficient to induce Mcl-1 expression in these cells.

We further investigated whether c-MET signaling is required for VEGF_165 _induction of Mcl-1. Indeed, VEGF_165 _only induced Mcl-1 expression in ARCaP_M _cells transfected with control siRNA, not in those expressing c-MET siRNA (Figure 4c). PHA-665752, a c-MET selective inhibitor [28], was used to treat ARCaP_M _cells prior to addition of VEGF_165_. PHA-665752 significantly attenuated VEGF_165 _induction of Mcl-1 in ARCaP_M _cells (Figure 4d). These data indicated that c-MET signaling is required for VEGF regulation of Mcl-1 in PCa cells.

### VEGF induces c-MET activation by a NRP1-dependent mechanism in PCa cells

**Figure 5:**
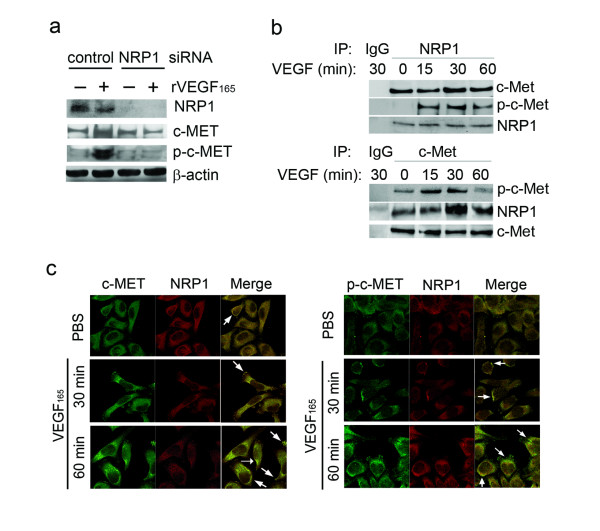
**VEGF_165 _induces c-MET activation through a NRP1-dependent mechanism**. (a) Effects of NRP1 depletion on VEGF_165_-mediated c-MET phosphorylation. ARCaP_M _cells were transfected with NRP1 siRNA or control siRNA for 48 h, serum-starved overnight, then treated with VEGF_165 _(10 ng/ml) for 60 min. (b) Immunoprecipitation assay of NRP1-c-MET interaction. Serum-starved ARCaP_M _cells were treated with VEGF_165 _(10 ng/ml) for the indicated times, and immunoprecipitated with anti-NRP1 (upper), or anti-c-MET (low) antibody. (c) Co-localization of NRP1 and c-MET or p-c-MET. Serum-starved ARCaP_M _cells were treated with VEGF_165 _(10 ng/ml) or PBS for the indicated times. Immunofluorescence staining of NRP1, c-MET or p-c-MET was performed and visualized by confocal microscopy. Arrows indicate co-localization of NRP1 and c-MET or p-c-MET.

c-MET activation involves phosphorylation of several tyrosine residues including those at positions 1230, 1234, and 1235 [29]. To assess whether VEGF_165 _could induce c-MET activation, and whether this process was mediated by NRP1, ARCaP_M _cells were transiently transfected with NRP1 siRNA or control siRNA before VEGF_165 _treatment. VEGF_165 _induced rapid phosphorylation of c-MET at Tyr1230/1234/1235 residues in ARCaP_M _cells transfected with control siRNA, but this effect was significantly attenuated by expression of NRP1 siRNA. Expression of total c-MET protein was not affected by siRNA treatment (Figure 5a). These data indicated that VEGF_165 _activated c-MET signaling independent of HGF, and NRP1 may be indispensible in this process.

### VEGF promotes interaction between NRP1 and c-MET in PCa cells

To explore whether VEGF could induce physical interaction between NRP1 and c-MET, an immunoprecipitation assay was performed in ARCaP_M _cells treated with VEGF_165 _for varying times. First, endogenous NRP1 protein was immunoprecipitated (Figure 5b, upper). There was a constitutive association between c-MET and NRP1 in the absence of VEGF_165_. Upon VEGF_165 _treatment, presence of c-MET in the NRP1 immunoprecipitates increased at 30 min and returned to baseline at 60 min. Phosphorylated c-MET (p-c-MET) significantly increased at 15 min following VEGF_165 _treatment, reached a peak at 30 min and slightly decreased in 60 min. Reciprocal immunoprecipitation with anti-c-MET antibody confirmed an association of NRP1 with c-MET in the absence of VEGF_165_. The presence of NRP1 and p-c-MET in the protein complex exhibited a similar time course following VEGF_165 _stimulation, with the peak at 30 min (Figure 5b, low).

**Figure 6:**
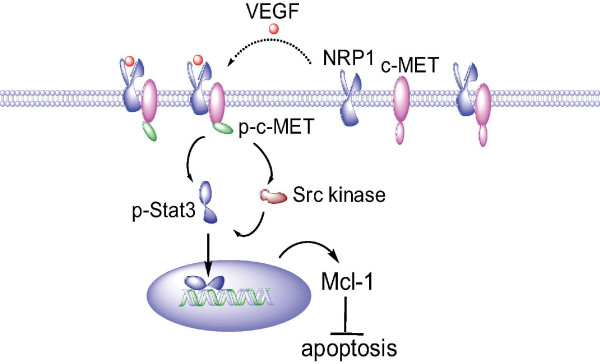
**A proposed model for VEGF_165 _regulation of Mcl-1 in PCa cells**. NRP1 may be constitutively associated with c-MET on plasma membrane. VEGF_165 _engagement recruits c-MET into the protein complex and promotes its interaction with NRP1, thereby enhancing phosphorylation of c-MET. Src kinase-Stat3 signaling is subsequently activated, resulting in nuclear translocation of p-Stat3 and activation of Mcl-1 expression. Increased intracellular Mcl-1 protects PCa cells from apoptosis.

Confocal microscopy was performed to determine whether VEGF_165 _promotes NRP1 interaction with c-MET and activation of c-MET in ARCaP_M _cells. NRP1 and c-MET were found to be constitutively associated on plasma membrane, with the intensity of co-localization further increased at 30 min upon VEGF_165 _treatment (Figure 5c, left panel). Notably, there was a more significant increase in the intensity of co-localization of NRP1 and p-c-MET following VEGF_165 _stimulation (Figure 5c, right panel). The data independently supported a mechanism that NRP1 may be constitutively associated with c-MET on plasma membrane. Upon VEGF_165 _binding, NRP1 may further recruit c-MET and facilitate its activation, subsequently transmitting VEGF_165 _signal (Figure 6).

### Role of Src kinases and signal transducers and activators of transcription 3 (Stat3) in VEGF induction of Mcl-1 in PCa cells

**Figure 7:**
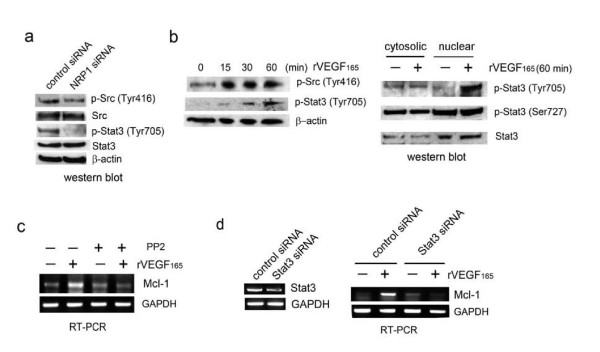
**Src kinase-Stat3 signaling mediates VEGF_165 _induction of Mcl-1 expression in ARCaP_M _cells**. (a) Effects of NRP1 inhibition on phosphorylation of Src kinases and Stat3. ARCaP_M _cells were transfected with NRP1 siRNA or control siRNA for 72 h. (b) Effects of VEGF_165 _on the phosphorylation of Src kinases and Stat3. ARCaP_M _cells were treated with VEGF_165 _(10 ng/ml) for the indicated time, and immunoblotting was performed on total lysates (left) and nuclear extracts and cytoplasmic proteins (right). (c) Effects of Src kinases on VEGF_165 _regulation of Mcl-1. ARCaP_M _cells were treated with PP2 (10 μM) or DMSO for 2 h before treatment with VEGF_165 _or PBS. (d) Effects of Stat3 depletion on VEGF_165 _induction of Mcl-1. ARCaP_M _cells were transfected with Stat3 siRNA or control siRNA (80 nM) for 48 h, serum-starved overnight, then treated with VEGF_165 _(10 ng/ml) or PBS for 2 h.

Activation of the Src kinase-Stat3 pathway is an important downstream event in c-MET signaling [30,31] (refer to Figure 6). Recently, a Stat3 *cis*-element was identified in human Mcl-1 promoter [32]. We investigated whether the Src kinase-Stat3 pathway is a downstream component in NRP1 signaling in ARCaP_M _cells (Figure 7a). Indeed, expression of NRP1 siRNA in ARCaP_M _cells significantly inhibited phosphorylation of Src kinases at Tyr416 (p-Src), as well as activation of Stat3 at Tyr705 (p-Stat3), without altering expression of endogenous Src kinases and Stat3.

Next we examined whether VEGF induces activation of Src kinase-Stat3 signaling in ARCaP_M _cells (Figure 7b). VEGF_165 _rapidly induced expression of both p-Src (Tyr416) and p-Stat3 (Tyr705) in a time-dependent manner in ARCaP_M _cells, with the peak at 60 min. Significantly, VEGF_165 _promoted rapid intracellular translocation of p-Stat3 (Tyr705) from the cytoplasm to the nucleus, indicating activation of Stat3-dependent gene expression. By contrast, nuclear presence of p-Stat3 (Ser727), which has been associated with HGF-induced Mcl-1 expression in primary human hepatocytes [27], was not affected. These data indicated that Src kinase-Stat3 pathway activation may be an important event following VEGF_165 _stimulation.

Finally, we assessed the role of Src kinase-Stat3 signaling in VEGF_165 _regulation of Mcl-1. PP2, a selective inhibitor of Src kinases [33], was used to treat ARCaP_M _cells before VEGF_165 _stimulation. PP2 treatment effectively abrogated VEGF_165 _induction of Mcl-1 (Figure 7c). Similarly, Mcl-1 mRNA expression was rapidly induced by VEGF_165 _in ARCaP_M _cells transfected with control siRNA, but not in the cells expressing Stat3 siRNA (Figure 7d). Collectively these data suggested that Src kinase-Stat3 signaling may be required for VEGF induction of Mcl-1 in PCa cells (Figure 6).

### NRP1 overexpression and c-MET activation are positively associated with human PCa progression and bone metastasis

**Figure 8:**
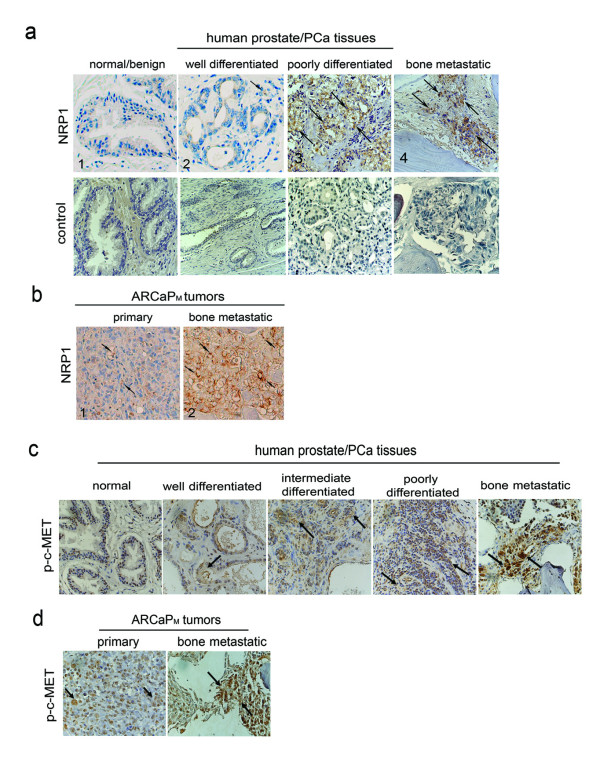
**Expression of NRP1 and p-c-MET is associated with bone metastatic status of human PCa specimens and the ARCaP_M _model**. IHC analyses of NRP1 expression in human normal/benign, cancerous and metastatic prostatic tissue specimens (a) and primary and bone metastatic tissue specimens from ARCaP_M _model (b), and of p-c-MET expression in human PCa progression (c) and ARCaP_M _xenografts (d). Arrows indicate positively-stained cells.

To validate the clinical significance of NRP1-c-MET signaling in PCa progression, and avoid the potential bias from using human PCa cell lines, IHC analyses were performed to determine the expression of NRP1 and p-c-MET in human PCa tissue specimens. Prostatic tissue specimens of normal/benign glands, primary and bone metastatic tumors were analyzed. NRP1 expression was increased from normal/benign glands (0/5) or well-differentiated cancer (1/5) to poorly-differentiated cancers (5/5) and bone metastatic tissues (5/5) (Figure 8a). NRP1 staining was also determined in tumor specimens from the ARCaP_M _xenograft model in which ARCaP_M _cells were inoculated into athymic mice orthotopically, resulting in skeletal metastases with a short latency [21]. Consistently, NRP1 expression was significantly greater in bone metastatic tumors than in primary tumors (Figure 8b).

We and others have reported that c-MET overexpression is positively associated with PCa progression [34,35]. As shown in Figure 8c, p-c-MET was expressed at a low level in normal human prostatic tissue, but increased significantly from well-differentiated and intermediate to poorly-differentiated primary PCa. Importantly, bone metastatic PCa specimens displayed a higher expression of p-c-MET than primary PCa. p-c-MET expression was also remarkably increased in bone metastatic ARCaP_M _tumors (Figure 8d).

## Discussion

Aberrant overexpression of Mcl-1 has been associated with poor prognosis and resistance to chemotherapy in a variety of human cancers [36]. Sensitizing tumor cells to apoptosis induction by selectively targeting Mcl-1, in combination with conventional chemotherapy, has emerged as an attractive therapeutic strategy [3,37]. In this study, we presented evidence that elevated Mcl-1 expression is associated with clinical PCa progression, particularly bone metastasis. We further showed that activation of VEGF-NRP1-c-MET signaling is responsible for Mcl-1 expression, which may confer survival advantages allowing PCa cells to evade apoptosis and progress towards invasive states (Figure 6).

Only limited information are available on Mcl-1 expression profile in PCa. An early study found that Mcl-1 expression was increased at tumors (81%) compared with only 38% in PIN. The percentage of Mcl-1-positive cells was typically higher in Gleason grade 8-10 tumors and metastasis than lower grade tumors, but there was no significant difference in Mcl-1 immunointensity between high grade tumors (47%) and metastasis in lymph node (38%) and bone (50) [5]. Our present study confirmed elevated Mcl-1 expression in high grade (≥ 7) PCa, though the difference between Gleason score 7 and 8-10 tumors is not statistically significant. Intriguingly, our results revealed a remarkable increase in Mcl-1 immunointensity in bone metastasis compared to primary tumors, indicating that Mcl-1 overexpression is positively correlated to PCa progression towards metastatic status in clinical situation.

Accumulating evidence suggests that the function of VEGF in tumor progression may not be limited to angiogenesis [38]. Numerous tumor cells express significant levels of VEGF-Rs, which could engage VEGF and initiate multiple signaling responses involved in cell proliferation, survival and migration. VEGF autocrine signaling confers a degree of self-sufficiency that may be crucial to metastasis as the microenvironment becomes increasingly hostile. Nonetheless, it remains controversial whether VEGF has significant autocrine effects in PCa cells, since the "classical" VEGF-Rs, i.e., VEGF-R1 and -R2, are undetectable in most established PCa cell lines [11,39,40]. Previously we reported that serum VEGF is positively associated with bone metastatic status in PCa patients, and recapitulated this close association in the ARCaP model [18]. In this study, we investigated whether VEGF could affect PCa cell behavior in an autocrine manner. Intriguingly, VEGF_165 _was found to be capable of inducing Mcl-1 expression within a non-saturating range, suggesting it may act as a survival factor in PCa cells. Moreover, NRP1 was found to be highly expressed by PCa cell lines and displayed a positive association with invasiveness, suggesting that it may be the primary receptor responsible for VEGF autocrine effects in PCa cells. Gene transfer experiments supported an indispensible role of NRP1 in mediating VEGF_165 _regulation of Mcl-1 in metastatic PCa cells. Importantly, a positive association between NRP1 expression and *in vivo *bone metastatic potential was found in ARCaP_M _xenografts and further confirmed in clinical PCa specimens. These data collectively support a novel role of NRP1 in PCa progression and metastasis, presumably by transmitting the VEGF autocrine survival signal and enabling PCa cells to evade apoptosis.

NRP1 is not a typical "signaling receptor" since it lacks sequences predicted to have kinase receptor activities [24]. Direct interaction between NRP1 and VEGF-Rs or plexin A is essential to its function in prioritizing differential signals from VEGF_165 _or semaphorin 3 in endothelial cells and nerve cells [24]. Recent studies found that the NRP1 intracellular domain, especially the C-terminal three amino acids (SEA-COOH), may be required for the interaction between NRP1 and its binding partners, including NRP1-interacting protein (NIP; or RGS-GAIP-interacting protein, GIPC) and VEGF-R2 [41,42]. However, the molecular effects and mechanism for NRP1-mediated signaling in tumor cells, especially in the absence of VEGF-Rs, remain elusive. It has been proposed that NRP1 may store or sequester VEGF_165 _and attract endothelial cells towards tumor, contributing to angiogenesis *via *juxtacrine or paracrine mechanisms [9,17]. In this study, we investigated whether NRP1 is required for VEGF_165 _autocrine signaling in PCa cells lacking VEGF-Rs. We presented evidence that NRP1 and c-MET are physically associated on plasma membrane, and in response to VEGF_165 _stimulation, their interaction may significantly facilitate further recruitment and activation of c-MET. Though the structural and molecular basis for interaction between the two receptors needs to be further investigated, this study indicates for the first time that c-MET activity can be modulated by VEGF, and the NRP1-c-MET interaction may be a critical component in transmitting the VEGF survival signal in PCa cells. It may be plausible to assign c-MET phosphorylation as an indicator for activation of NRP1 autocrine signaling in tumor cells. Using human PCa tissue specimens as the "gold standard", we observed that NRP1 and p-c-MET were both significantly increased with progression of primary PCa, and further elevated in bone metastatic PCa, suggesting that activation of NRP1-c-MET signaling may be positively associated with clinical PCa progression. It is worth noting that these results need cautious interpretation since a limited number of patient specimens was examined, and the polyclonal antibody against p-c-MET has not been fully validated for immunohistochemical analysis in tissue sections.

In human primary hepatocytes, HGF transcriptionally induced Mcl-1 expression, but not Bcl-2 or Bcl-x(L) [27]. Our studies found that, however, HGF treatment did not significantly affect Mcl-1 protein expression in ARCaP_M _cells, suggesting HGF activation of c-MET signaling may not be sufficient to increase Mcl-1 expression. We postulated that VEGF_165 _may utilize a different mechanism to regulate Mcl-1 expression in PCa cells. An important observation supporting this hypothesis is that VEGF_165 _only rapidly induced Stat3 phosphorylation at Tyr705, but not altering Ser727 phosphorylation in ARCaP_M _cells. In hepatocytes, however, HGF triggered Ser727, but not Tyr705, phosphorylation of Stat3 [27]. It will be intriguing to investigate the role of NRP1-c-MET interaction in differentiating extracellular ligands, i.e., VEGF_165 _or HGF, and activating distinct downstream cascades leading to Mcl-1 expression in a highly cell context-dependent fashion.

Mcl-1 is a protein with very short half-life and is highly regulated at multiple levels in response to survival and differentiation signals. Rapid turnover of Mcl-1 protein can be initiated by stress (such as serum withdrawal) through caspase-mediated and proteasome-dependent degradation in tumor cells [3]. Previous study found that VEGF protects multiple myeloma cells against apoptosis by up-regulating Mcl-1 protein in a time-dependent manner, peaking at 6 h and returning to baseline after 24 h; change in Mcl-1 mRNA expression was not reported [43]. In ARCaP_M _cells, however, VEGF_165 _treatment rapidly induced Mcl-1 mRNA expression and significantly increased Mcl-1 protein level at 72 h. Though whether VEGF_165 _may also regulate Mcl-1 expression at other levels needs to be further investigated, the data presented here suggest that transcriptional activation, presumably mediated by Stat3, is an important mechanism for VEGF_165 _induction of Mcl-1 in PCa cells.

Interrupting NRP1 functions with mimetic peptides and monoclonal antibodies is being developed in xenograft models of human cancers [44,45]. NRP1 monoclonal antibody has been shown to effectively inhibit tumor vascular remodeling, rendering vessels more susceptible to anti-VEGF therapy [45]. If the VEGF_165_-NRP1-c-MET pathway is required for Mcl-1 expression and survival in PCa cells, it would be intriguing to evaluate whether targeting NRP1 could interrupt both NRP1-c-MET signaling in tumor cells (survival) and NRP1-VEGF-Rs signaling in endothelial cells (angiogenesis). Inhibition of NRP1 signaling may be a promising strategy alone or in combination with other therapeutic approaches for treating PCa.

## Methods

### Cell culture

Human PCa cell lines ARCaP_M_, ARCaP_M_-C2, PC3, LNCaP, C4-2 and C4-2B, were routinely maintained in T-medium (Invitrogen, Carlsbad, CA) with 5% fetal bovine serum (FBS). ARCaP_M_-C2 subclone was derived from ARCaP_M _bone metastatic tissues as described previously [21,22]. Where specified, ARCaP_M _cells were serum-starved overnight, and treated with recombinant human VEGF_165_, VEGF_121_, HGF (R&D Systems, Minneapolis, MN), c-MET inhibitor PHA-665752 (Calbiochem, San Diego, CA), or Src kinase inhibitor (PP2) (Calbiochem) in serum-free T-medium. HUVEC (The American Type Culture Collection, Manassas, VA) were maintained in endothelial basal growth medium (EBM-2) with 2% FBS. Cell proliferation was measured using the CellTiter 96 AQ proliferation assay according to the manufacturer's instructions (Promega, Madison, WI). Viable cells were counted in triplicate using a hemacytometer and trypan blue staining.

### Immunohistochemical analysis

IHC staining of NRP1 and p-c-MET on human normal/benign prostatic glands, well- and poorly-differentiated primary PCa and bone metastatic PCa tissue specimens was performed as described previously [18] using goat anti-NRP1 antibody (C-19, Santa Cruz Biotechnology, Santa Cruz, CA; 1:100) and rabbit anti-p-c-MET (Tyr1230/1234/1235) antibody (44888G, Invitrogen; 1:100). IHC analysis of Mcl-1 expression was performed on a human PCa progression tissue microarray specimen (US BioMax, Inc., Rockville, MD) using rabbit anti-Mcl-1 antibody (S-19, Santa Cruz Biotechnology, 1:150). Matching normal serum was used as negative control. All IHC common reagents were obtained from Dako (Carpinteria, CA). Positive expression of NRP1, p-c-MET and Mcl-1 was defined as >15% positive staining in cell population.

### Transfection

The vector harboring NRP1 cDNA (pCMV-NRP1) and the control (pCMV-XL4) (Origene, Rockville, MD) were transfected into ARCaP_M _cells for 48-72 h using lipofectamine 2000 (Invitrogen). Small interfering RNA (siRNA) nucleotides were transfected into ARCaP_M _cells according to the manufacturer's instructions. VEGF siRNA, Mcl-1 siRNA, Stat3 siRNA and control siRNA-A were purchased from Santa Cruz Biotechnology. NRP1 *silencer*^® ^select validated siRNA and *silencer*^® ^select control siRNA^#^1 were obtained from Ambion, Inc., (Austin, TX). c-MET ON-TARGET *plus *siRNA and ON-TARGET *plus *siRNA control were purchased from Dharmacon, Inc, (Chicago, IL).

### Western blot analysis

Total cell lysates were prepared using radioimmunoprecipitation (RIPA) buffer (Santa Cruz Biotechnology). Nuclear proteins were extracted using a Novagen kit (EMD Biosciences, San Diego, CA). Immunoblotting analysis followed standard procedure [18] with anti-Stat3, anti-p-Stat3 (Ser705), anti-Src and anti-p-Src (Tyr416) (Cell Signaling, Danvers, MA); anti-p-Stat3 (Tyr705) (Upstate, Charlottesville, VA); anti-Mcl-1, anti-NRP1 (C-19), anti-NRP2, anti-VEGF-R2, and anti-c-MET (Santa Cruz Biotechnology); anti-p-c-MET (Tyr1230/1234/1235) (Invitrogen); and anti-β-actin (Sigma, St. Louis, MO).

### RT-PCR

Total RNA was prepared with Qiagen RNeasy Kit (Valencia, CA), and RT-PCR was performed using the SuperScriptIII^® ^One-Step RT-PCR kit (Invitrogen) following the manufacturer's protocol. The specific primer pairs are: 5'-GAGGAGGAGGAGGACGAGTT-3' (forward) and 5'-GTCCCGTTTTGTCCTTACGA-3' (reverse) (for Mcl-1); 5'-AGGACAGAGACTGCAAGTATGAC-3' (forward) and 5'-AACATTCAGGACCTCTCTTGA-3' (reverse) (for NRP1). The primers for VEGF, glyceraldehyde-3-phosphate dehydrogenase (GAPDH) [18] and Stat3 [46] were described previously. The primer pairs for human VEGF-R1, VEGF-R2 and NRP2 were purchased from R&D Systems.

### ELISA

Subconfluent PCa cells were cultured in serum-free T-medium for 72 h before conditioned medium was collected. VEGF concentrations were analyzed using a Quantikine ELISA kit (R&D Systems) and normalized with total protein concentrations in CM.

### Immunofluorescence and confocal imaging

Immunofluorescence was performed as described previously [47]. Goat anti-NRP1 antibody (C-19), rabbit anti-c-MET antibody (C-12, Santa Cruz Biotechnology), or rabbit anti-p-c-MET (Tyr1230/1234/1235) antibody was incubated with subconfluent ARCaP_M _cells at 4°C overnight. Either anti-goat Alexa Fluor^® ^546 or anti-rabbit Alexa Fluor^® ^488 secondary antibody (Invitrogen) was used at a dilution of 1:500. Cells were imaged on a Zeiss LSM 510 META [47]. In all cases, either a 63× or 100× Zeiss Plan-Apo oil objective was used (numerical aperture of 1.3 and 1.4, respectively). All images had contrast expansion performed in Adobe Photoshop.

### Immunoprecipitation

The Immunoprecipitation Starter Pack (GE healthcare Bio-Sciences Corp., Piscataway, NJ) was used according to the manufacturer's instructions. Total lysates (1 mg) were immunoprecipitated with rabbit anti-c-MET antibody (C-12), rabbit anti-NRP1 antibody (H-286, Santa Cruz Biotechnology), or normal rabbit IgG (R&D Systems). Protein A/G Sepharose 4 Fast Flow beads were added to precipitate proteins, then washed and eluted. The samples were further processed for western blot analysis.

### Apoptosis analysis

Cells were stained with an Annexin V-PE apoptosis detection kit (BD Biosciences, San Jose, CA) following the manufacturer's protocol, and measured using a fluorescence-activated cell sorting (FACS) caliber bench-top flow cytometer (Becton Dickinson, Franklin Lakes, NJ). The data were analyzed using FlowJo software (Tree Star, Inc., Ashland, OR).

### Data analysis

Significance levels for comparisons of Mcl-1 expression in different Gleason score PCa were calculated by using the 2-sample *t *test. Treatment effects were evaluated using a two-sided Student's *t *test. All data represent three or more experiments. Errors are S.E. values of averaged results, and values of *p *< 0.05 were taken as a significant difference between means.

## Competing interests

The authors declare that they have no competing interests.

## Authors' contributions

SZ performed western blotting, immunoprecipitation, immunofluorescence confocal microscopy, and gene transfer experiments (siRNA and cDNA expression). HEZ established the ARCaP PCa progression model, provided human PCa tissue specimens, designed and performed the immunohistochemical staining of NRP1 and p-c-MET in ARCaP tumors and human PCa specimens. AOO provided human PCa tissue specimens and evaluated expression of NRP1 and p-c-MET. SF evaluated expression of Mcl-1 in PCa tissue microarray specimens. ZC performed statistical analyses. XY and SI performed cell culture and preparation of proteins and RNA samples. RW contributed to the establishment of the ARCaP animal models. FFM and LWKC participated in discussion and manuscript preparation. LWKC provided grant supports for this study. DW designed experiments and drafted manuscript. All authors read and approved the final version of this manuscript.

## Supplementary Material

Additional file 1 **Figure S1**. (a) Endogenous Mcl-1 expression in the lineage-related LNCaP, C4-2 and C4-2B cells, as determined by RT-PCR and western blotting analyses. (b) ELISA of VEGF levels in conditioned media of LNCaP and C4-2 cells, shown as relative VEGF concentrations normalized by total protein concentrations of the CM. (c) Effects of recombinant VEGF_121 _on the proliferation of ARCaP_M _cells. 1 × 10^3^cells were seeded in 96-well plates for 24 h, serum-starved overnight, and cultured in the absence or presence of varying concentrations of rVEGF_121 _for 72 h. MTS assay was then performed. (d) Effects of VEGF_165 _on Mcl-1 expression in LNCaP cells. Subconfluent LNCaP cells were serum-starved overnight, and incubated for 72 h in the presence of VEGF_165 _(10 ng/ml) or PBS. Western blotting was performed. (e) Effects of VEGF_121 _on Mcl-1 expression in ARCaP_M _cells. Subconfluent ARCaP_M _cells were serum-starved overnight, and incubated for 72 h in the presence of VEGF_121 _(10 ng/ml) or PBS. Western blotting was performed. (f) Expression of endogenous NRP1 in LNCaP, C4-2 and C4-2B cells, as determined by RT-PCR and western blotting analyses. (g) Nuclear expression of c-MET and p-c-MET in ARCaP_M _and ARCaP_M_-C2 cells. TATA binding protein (TBP) was used as internal control of nuclear proteins. Click here for file
