# Supplementary figures and images for: Vascular endothelial growth factor regulates myeloid cell leukemia-1 expression through neuropilin-1-dependent activation of c-MET signaling in human prostate cancer cells
Source: Mol Cancer. 2010 Jan 19;9:9. doi: 10.1186/1476-4598-9-9 (PMC2820018; doi:10.1186/1476-4598-9-9)

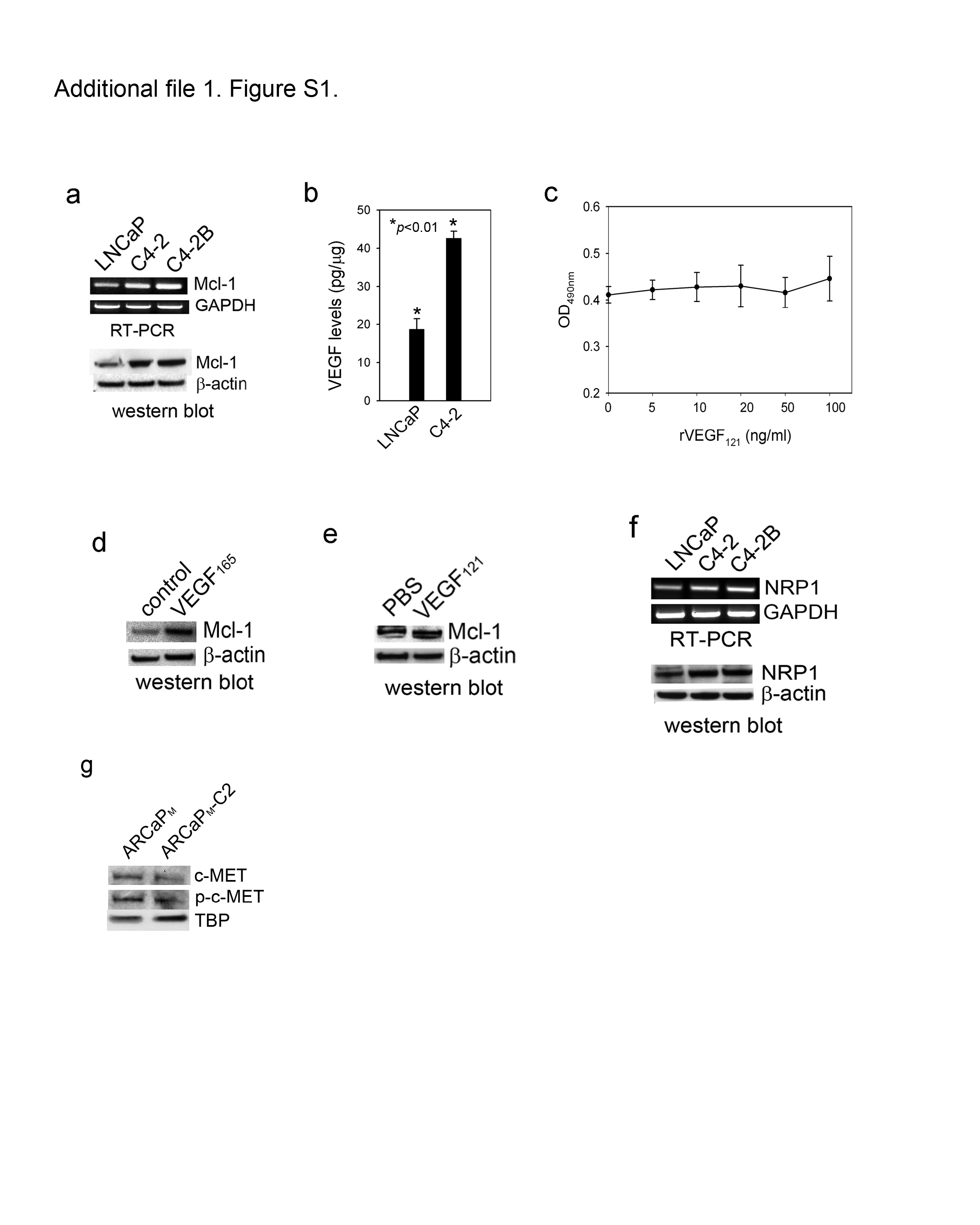

Supplement: Additional file 1 — Figure S1. (a) Endogenous Mcl-1 expression in the lineage-related LNCaP, C4-2 and C4-2B cells, as determined by RT-PCR and western blotting analyses. (b) ELISA of VEGF levels in conditioned media of LNCaP and C4-2 cells, shown as relative VEGF concentrations normalized by total protein concentrations of the CM. (c) Effects of recombinant VEGF121 on the proliferation of ARCaPM cells. 1 × 10^3^cells were seeded in 96-well plates for 24 h, serum-starved overnight, and cultured in the absence or presence of varying concentrations of rVEGF121 for 72 h. MTS assay was then performed. (d) Effects of VEGF165 on Mcl-1 expression in LNCaP cells. Subconfluent LNCaP cells were serum-starved overnight, and incubated for 72 h in the presence of VEGF165 (10 ng/ml) or PBS. Western blotting was performed. (e) Effects of VEGF121 on Mcl-1 expression in ARCaPM cells. Subconfluent ARCaPM cells were serum-starved overnight, and incubated for 72 h in the presence of VEGF121 (10 ng/ml) or PBS. Western blotting was performed. (f) Expression of endogenous NRP1 in LNCaP, C4-2 and C4-2B cells, as determined by RT-PCR and western blotting analyses. (g) Nuclear expression of c-MET and p-c-MET in ARCaPM and ARCaPM-C2 cells. TATA binding protein (TBP) was used as internal control of nuclear proteins. [file 1476-4598-9-9-S1.TIFF]
